# Prognostic models in male breast cancer

**DOI:** 10.1007/s10549-016-3991-9

**Published:** 2016-09-26

**Authors:** Carmen C. van der Pol, Miangela M. Lacle, Arjen J. Witkamp, Robert Kornegoor, Hui Miao, Christine Bouchardy, Inne Borel Rinkes, Elsken van der Wall, Helena M. Verkooijen, Paul J. van Diest

**Affiliations:** 1 Department of Surgical Oncology, University Medical Center Utrecht, PO Box 85500, 3508 GA Utrecht, The Netherlands; 2 Department of Medical Oncology, University Medical Center Utrecht, Utrecht, The Netherlands; 3 Department of Radiology, University Medical Center Utrecht, Utrecht, The Netherlands; 4 Department of Pathology, University Medical Center Utrecht, Utrecht, The Netherlands; 5 Department of Pathology, Gelre Ziekenhuis, Apeldoorn, The Netherlands; 6 Saw Swee Hock School of Public Health, National University of Singapore, Singapore, Singapore; 7 Geneva Cancer Registry, Institute for Social and Preventive Medicine, Geneva University, Geneva, Switzerland

**Keywords:** Male breast cancer, Prognosis, Survival, Adjuvant! Online, NPI, Predict

## Abstract

**Purpose:**

Breast cancer in men is uncommon; it accounts for 1 % of all patients with primary breast cancer. Its treatment is mostly extrapolated from its female counterpart. Accurate predictions are essential for adjuvant systemic treatment decision-making and informing patients. Several predictive models are available for female breast cancer (FBC) including the Morphometric Prognostic Index (MPI), Nottingham Prognostic Index (NPI), Adjuvant! Online and Predict. The aim of this study was to examine and compare the prognostic performance of these models for male breast cancer (MBC).

**Methods:**

The population of this study consists of 166 MBC patients. The prognostic scores of the patients are categorized by good, (moderate) and poor, defined by the test itself (MPI and NPI) or based on tertiles (Adjuvant! Online and Predict). Survival according to prognostic score was compared by Kaplan–Meier analysis and differences were tested by logRank. The prognostic performances were evaluated with C-statistics. Calibration was done with the aim to estimate to what extent the survival rates predicted by Predict were similar to the observed survival rates.

**Results:**

All prediction models were capable of discriminating between good, moderate and poor survivors. *P*-values were highly significant. Comparison between the models using C-statistics (*n* = 88) showed equal performance of MPI (0.67), NPI (0.68), Adjuvant! Online (0.69) and Predict (0.69). Calibration of Predict showed overestimation for MBC patients.

**Conclusion:**

In conclusion, MPI, NPI, Adjuvant! and Predict prognostic models, originally developed and validated for FBC patients, also perform quite well for MBC patients.

**Electronic supplementary material:**

The online version of this article (doi:10.1007/s10549-016-3991-9) contains supplementary material, which is available to authorized users.

## Introduction

Breast cancer in men (MBC) is uncommon: it accounts for 1 % of all patients with early breast cancer [1]. Treatment protocols are largely extrapolated from the female counterparts. Accurate predictions are essential to be able to inform patients and advise on adjuvant systemic treatment following surgery for early breast cancer. A number of predictive models have been developed over time to assess prognosis in female breast cancer patients, including the Morphometric Prognostic Index (MPI) [2], Nottingham Prognostic Index (NPI) [3–6], Adjuvant! Online [7, 8] and Predict [9, 10]. The MPI was first described in 1985 and is based on the mitotic activity index (MAI), tumour size and lymph node status [1]. The NPI, first described in 1987, is based on tumour size, tumour grade and lymph node status [3]. Adjuvant! Online (www.adjuvantonline.com) and Predict (www.predict.nhs.uk) are online prediction tools that provide survival estimates and absolute individual adjuvant treatment benefit predictions. Adjuvant! Online calculates 10 years survival data. Predict calculates 5- as well as 10-year survival data. Adjuvant! Online was first described in 2001 [7]. Predict was developed in the United Kingdom, described in 2010, and was the first prognostication tool for early FBC patients to include HER-2-status and mode of detection [9]. All these prediction tools are based on data of FBC patients and it was unknown whether these outcome predictions would equally apply to MBC patients. The aim of this study was to investigate the validity and compare the predictive performance of these models, particularly concerning discrimination, in a relatively large group of male breast cancer patients.

## Patients and methods

### Study population

Demographic and clinical data and histopathological reports of all men surgically treated for invasive breast cancer between 1976–2010 were collected from four hospitals in The Netherlands (St. Antonius Hospital, Nieuwegein; *n* = 28, Diakonessenhuis Utrecht; *n* = 22, University Medical Centre Utrecht; *n* = 23, Laboratory for Pathology East Netherlands; *n* = 40), two hospitals in Germany (Paderborn; *n* = 8, and Koeln; *n* = 13) and from the population-based Geneva Cancer Registry (Switzerland, Geneva; *n* = 65). Hematoxylin and eosin (HE) slides of the Dutch and German male breast cancer patients were reviewed by three experienced observers (pathologists; PJvD, RK, AM) to confirm the diagnosis and to type and grade according to current standards. Pathology reports were used to extract age, tumour size and lymph node status. Patients with isolated tumour cells in the sentinel lymph nodes were regarded as lymph node negative. The original study group comprised 199 MBC patients. Follow-up data were available of 166 patients. For each patient, the data were calculated with the predictive models and compared with the actual 5-year overall survival time.

### Model calculations

Morphometric Prognostic Index was calculated using the following formula: MPI = 0.3341 × √(MAI) + 0.2342 × (tumour size in cm)−0.7654 × (lymph node status, pos = 1, neg = 2), where MAI is the mitotic activity index (number of mitosis per 1.6 mm^2^) [11].

According to the previously established threshold [2, 11], prognosis was categorized as “good” if the MPI was smaller than 0.60 and “poor” in case of MPI ≥ 0.60.

The Nottingham Prognostic Index (NPI) was calculated on the basis of the formula: NPI = [0.2 × *S*] + *N* + *G*, where *S* = the size in cm, *N* = the number of lymph nodes involved and *G* = tumour grade. The NPI defines three prognostic groups: ‘good’ for NPI ≤ 3.4, ‘moderate’ for 3.4 < NPI ≤ 5.4 and ‘poor’ for NPI > 5.4 (2)(3)(4).

### Adjuvant! Online

The web-based program www.adjuvantonline.com for breast cancer (Version 8.0) was used to calculate a prognosis for each individual patient. Age, comorbidity, ER status, tumour grade, tumour size, number of positive ipsilateral axillary nodes, adjuvant hormonal treatment and adjuvant chemotherapy were used to generate 10-year predictions of breast cancer-specific survival (BCSS) and disease-free survival (DFS), as well as the absolute benefit of adjuvant chemotherapy and hormonal therapy [7]. Due to no reliable data on comorbidity being available, ‘average for age’ was used. “Tamoxifen” was entered as hormonal therapy. For those who received adjuvant chemotherapy, individual information about the specific treatment was not available. Therefore, we defined the type of adjuvant therapy in line with the most commonly used treatments at the time of diagnosis. Data calculated by Adjuvant! Online are continuous, and therefore patients’ predicted overall survival probabilities were divided into tertiles to assure equal groups with standard normal distribution. Consequently, the prognosis calculated by Adjuvant! Online was classified as ‘good’ if the predicted 10-year survival probability was ≥70 %, ‘moderate’ if it was 45–70 % and ‘poor’ if the predicted 10-year survival was less than 45 %.

### Predict

The online Predict tool (www.predict.nhs.uk) uses age, mode of detection, tumour size, tumour grade, number of positive nodes, ER status, HER2 status, Ki67 status, adjuvant hormonal treatment, and adjuvant chemotherapy [9]. Breast cancer screening for men does not exist, and therefore ‘mode of detection’ was coded as ‘symptomatic’ for every patient. Due to the lack of individual information on the kind of chemotherapy used, the most commonly used at the time of diagnosis was filled out. The threshold for Ki67 was defined by the Predict tool itself: positive when more than 10 % of tumour cells stained positive. Prognostic groups were based on tertiles of the predicted 5-year overall survival probabilities (i.e. ≥90 %; ‘good’, 80–90 %; ‘moderate’, ≤80 %; ‘poor’) to assure equal groups with standard normal distribution, because data calculated by Predict are continuous variables.

### Statistics

For each model, Kaplan–Meier survival curves were plotted according to predicted prognostic groups and differences in the observed 5-year survival were tested with the LogRank test. Discrimination of the different models was estimated by means of the concordance index (C-index). A C-index of 1 indicates a perfect match of predicted and observed outcome. If the C-index is 0.5 the test does not predict any better than chance. Calibration could only be done for Predict, containing continuous variables and validated for 5-year survival. Observed and predicted outcomes were compared by use of a one-sample *t* test for proportions [13].

Statistical analyses were performed by means of IBM SPSS (version 20.0) and R.

## Results

The mean age of the 166 patients was 66.4 (range 32–92) years (Table 1). Most patients had T1 (55.4 %) or T2 (41 %) tumours, mostly ER positive (83.7 %) and 10.8 % unknown. HER2 was positive in 1.8 %, negative in 58.4 %, and 39.8 % unknown. Lymph node status was negative in 42.2 % of the cases (N0) and 1–3 lymph nodes with metastases in 19.9 % of the cases (N1), while 16.8 % had more than three lymph nodes positive (N2–3). 21.1 % of the axillary status was unknown. A total of 65 (39.2 %) patients underwent adjuvant radiotherapy and 69 (41.6 %) received hormonal treatment. Only 30 patients received adjuvant chemotherapy (18.1 %). Median survival was 4.6 years.

**Table 1:** Baseline characteristics of male breast cancer patients *N* = 166

Age		HER2	
Mean	66.4	Negative	97 (58.4)
≤65	74 (44.6)	Positive	3 (1.8)
>65	92 (55.4)	Unknown	66 (39.8)
T-status		MAI	
T1 = 0–2 (cm)	92 (55.4)	low < 10	49 (29.5)
T2 = 2, 1–5 (cm)	68 (41.10)	high ≥ 10	52 (31.3)
T3 > 5cm	3 (1.8)	Unknown	65 (39.2)
Unknown	3 (1.8)		
N-status		Ki67	
N0 = 0	70 (42.2)	Low < 10	80 (48.2)
N1 = 1–3	33 (19.9)	High ≥ 10	21 (12.6)
N2 = 4–9	18 (10.8)	Unknown	65 (39.2)
N3 ≥ 10	1 0 (6.0)	Radiotherapy	
Unknown	35 (21.1 )	No	94 (56.6)
		Yes	65 (39.2)
		Unknown	7 (4.2)
ER status		AHT1	
Positive	139 (83.7)	No	90 (54.2)
Negative	9 (5.4)	Yes	69 (41.6)
Unknown	18 (10.9)	Unknown	7 (4.2)
Grade		Chemotherapy	
1	25 (15.1)	No	129 (77.7)
2	69 (41.6)	Yes	30 (18.1)
3	50 (30.1)	Unknown	7 (4.2)
Unknown	22 (13.2)		

^1^Anti-hormonal treatment

Due to missing data, not every patient could be included in each predictive model. The MPI could be calculated for 88 patients, NPI for 124 patients (the same 88, plus 36 other patients), Adjuvant! Online for 130 (the same 124 and another six patients) and Predict for 158 patients (same patients as for Adjuvant! Online plus another 28 patients), (Table S1).

**Fig. 1:**
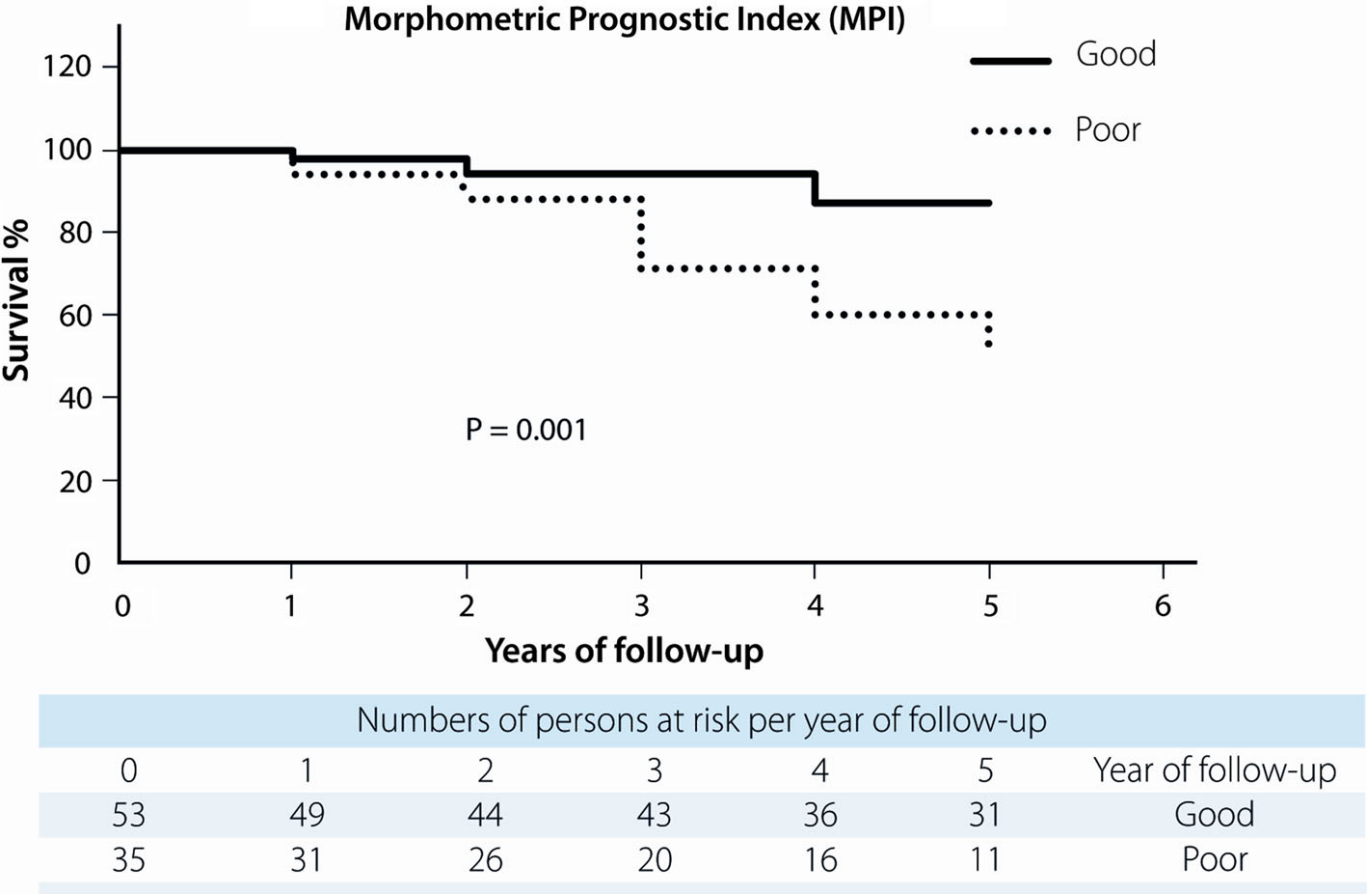
Survival curves for male breast cancer patients according to subgroups of the morphometric prognostic index

**Fig. 2:**
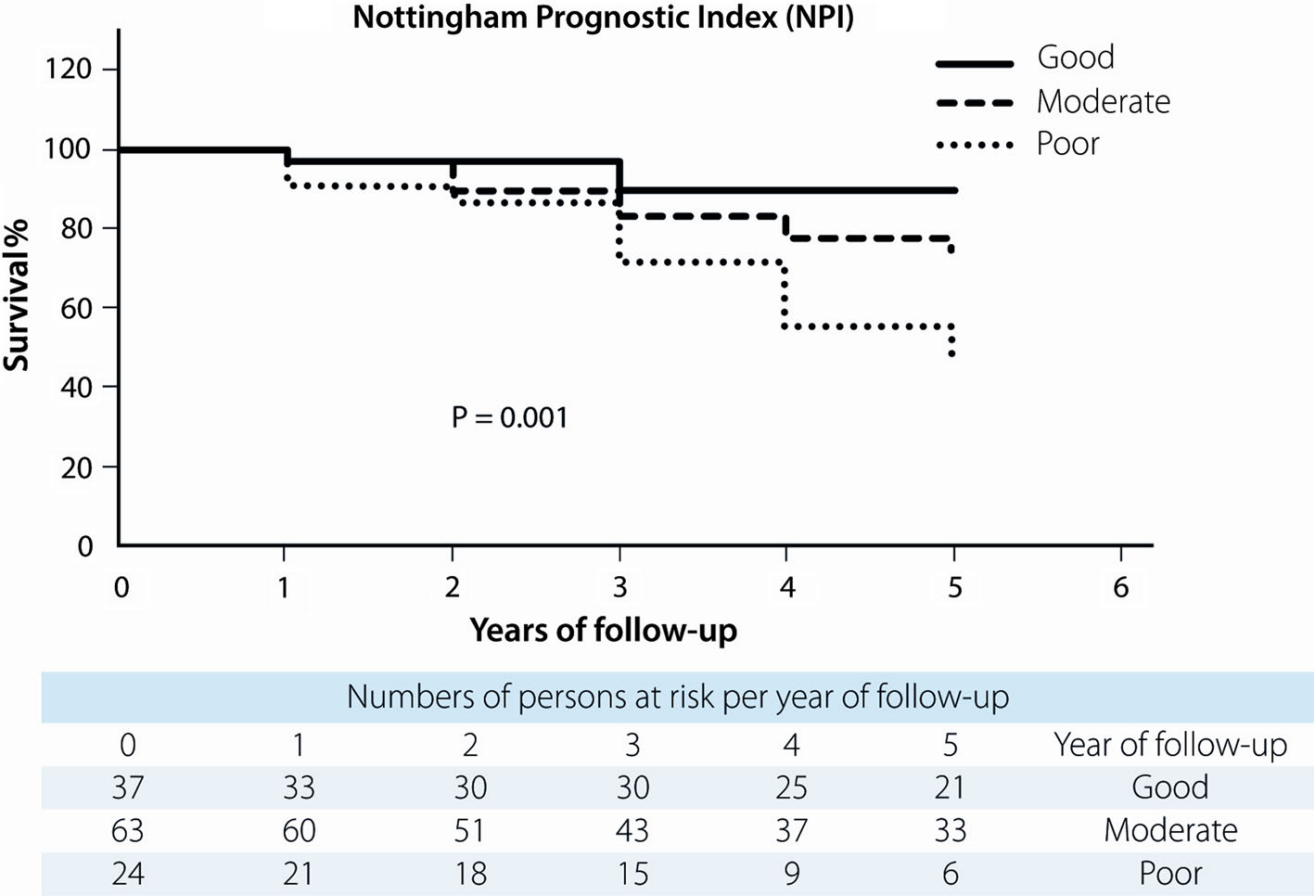
Survival curves for male breast cancer patients according to subgroups of the Nottingham prognostic index

**Fig. 3:**
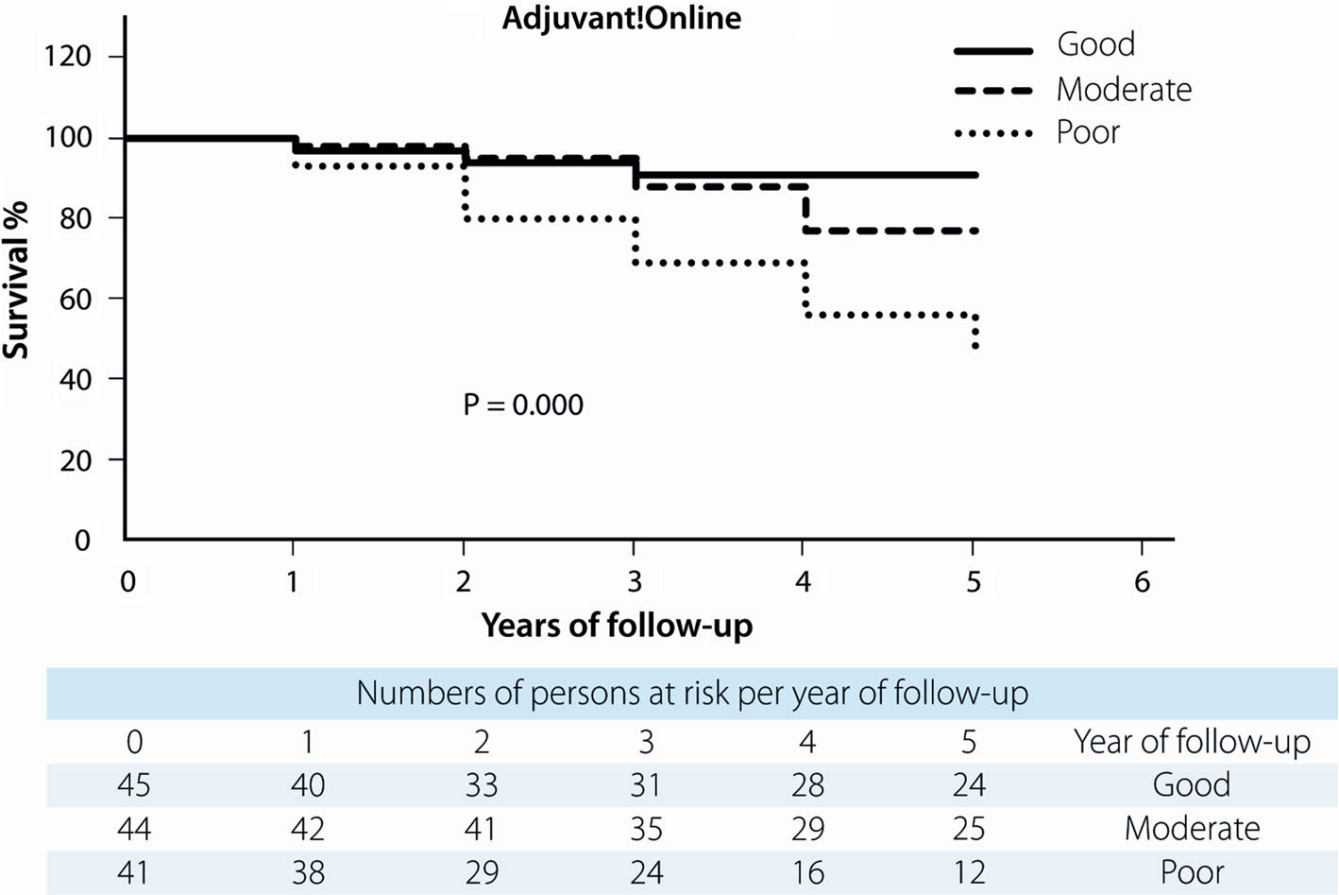
Survival curves for male breast cancer patients according to subgroups of Adjuvant! Online

**Fig. 4:**
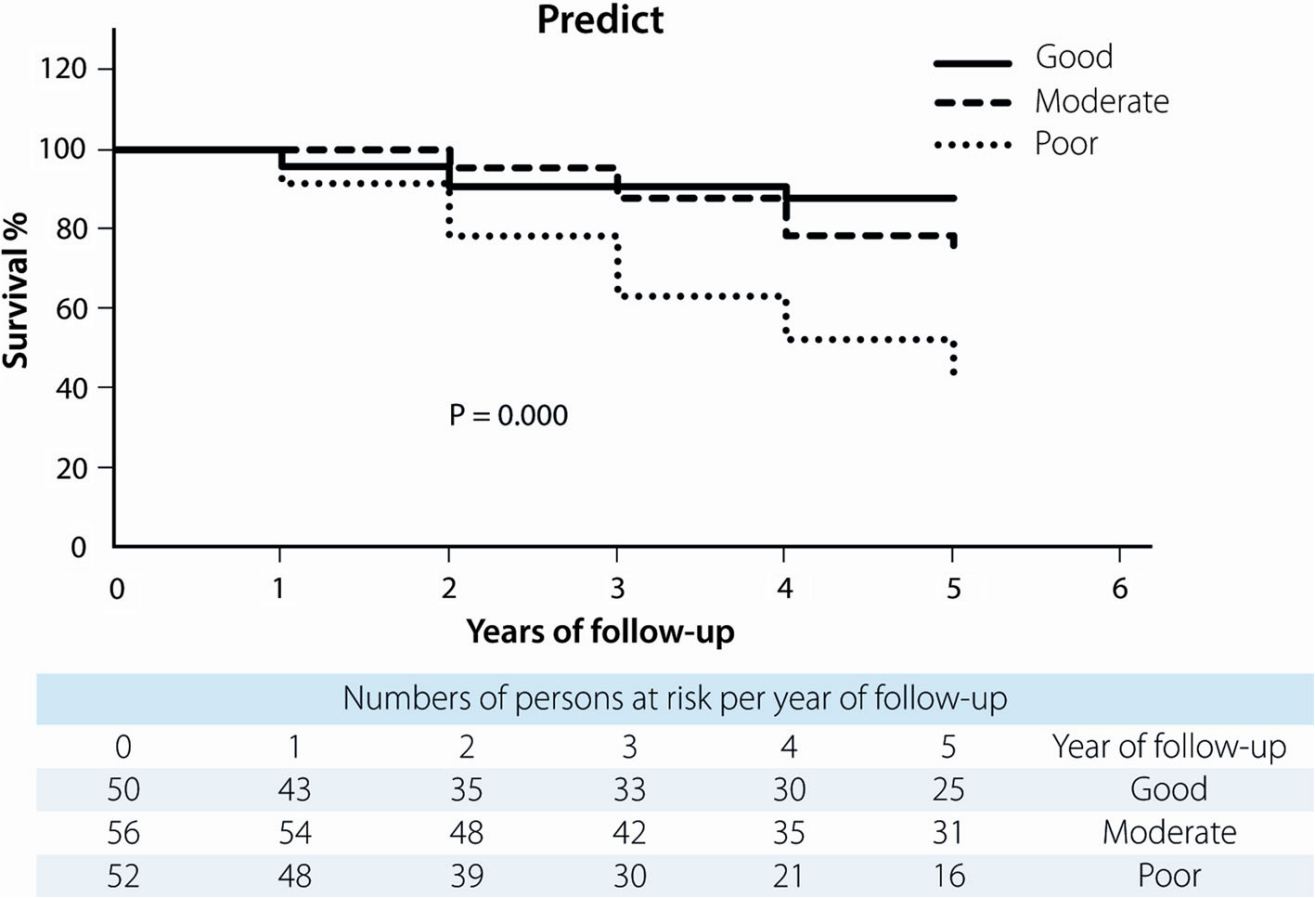
Survival curves for male breast cancer patients according to subgroups of predict

All four predictive models clearly and significantly separated MBC patients with a favourable and unfavourable outcome. MPI showed 87 % (95 % confidence interval (CI) 86.9–87.1) in 5-year survival for the “good” prognostic group and 51 % (95 % CI 50.8–51.2) for the “poor” prognostic group with *p* = 0.001. For NPI, this was 90 % (95 % CI 89.9–90.1) for the “good-” and 43 % (95 % CI 42.8–43.2) for the “poor” prognostic group with *p* = 0.001. Using Adjuvant! Online, this was 91 % (95 % CI 90.9–91.1) and 45 % (95 % CI 44.8–45.2), respectively, (*p* = 0.000), and according to Predict, 88 % (95 % CI 87.0–88.1) in the “good” prognostic group would be alive after 5 years and 42 % (95 % CI 41.9–42.1) in the “poor” prognostic group with a *p*-value of 0.000 (Figs. 1, 2, 3, 4). 5-year-observed survival probabilities were not significantly different for the good and moderate prognostic groups (NPI: *p* = 0.112, Adjuvant! Online *p* = 0.130 and Predict: *p* = 0.221). However, moderate and poor prognostic groups showed significantly different 5-year survival (NPI *p* = 0.014, Adjuvant! Online *p* = 0.003 and Predict *p* = 0.001) (Figs. 1, 2, 3, 4).

**Fig. 5:**
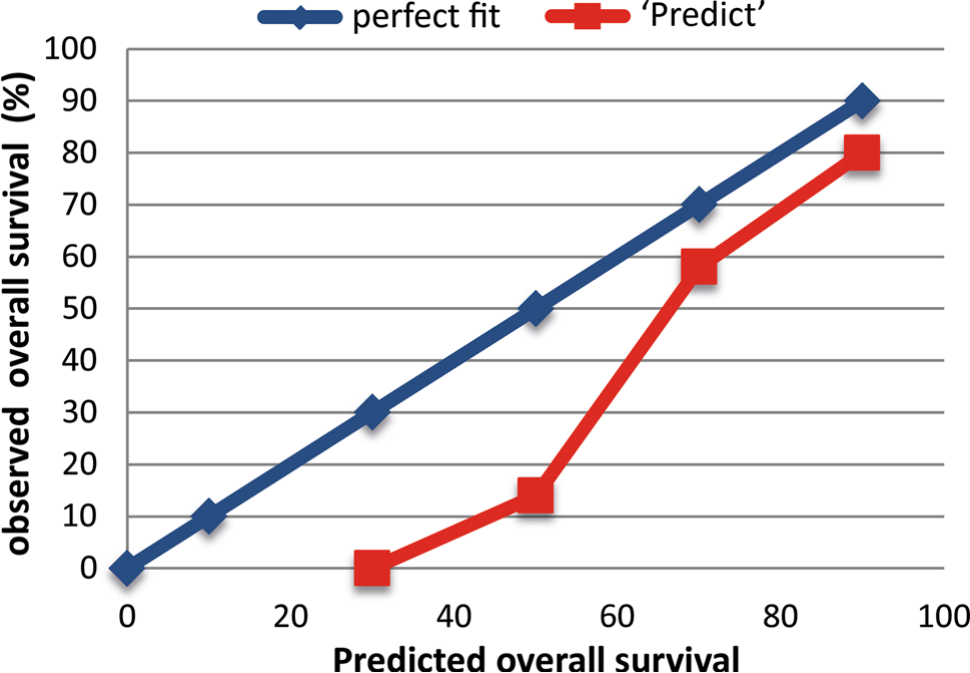
Calibration of predict 5 years

Discrimination between good and poor survivors was modest for all four models including the same 88 patients (Table S2). Including the maximal amount of patients per model showed similar results; C-index for MPI (*n* = 88) of 0.67 (95 % CI 0.58–0.77), for NPI (*n* = 124) 0.68 (95 % CI 0.60–0.76), for Adjuvant! Online (*n* = 130) 0.72 with 95 % CI 0.65–0.79 and 0.71 for Predict (*n* = 158) with 95 % CI 0.65–0.78 (Table S3). Calibration of Predict shows overestimation for this group of MBC patients (Fig. 5).

## Discussion

The present study compares the performance of prognostic tools such as the Morphometric Prognostic Index, Nottingham Prognostic Index, Adjuvant! Online and Predict in MBC patients. We found that these models, which were originally developed and validated for female breast cancer patients, perform quite well for MBC patients as well.

The MPI was first described in 1985 [11] and validated in several studies [2, 14–16]. The MPI is based on the mitotic activity index, tumour size and lymph node status. It is interesting to see that the MPI, which was developed much earlier and which does not include tumour grading, performed only slightly less well than Predict. Yet, it takes mitotic index into account which has been well established to be the most important constituent of grade [14, 16, 17] and a validated prognosticator of MBC [18].

The NPI was originally devised in 1978 by Blamey et al., formally described in 1982 [19] and validated in many studies [3, 5, 6, 12, 20]. It is a histopathological grading model that reflects tumour behaviour better than TNM because it takes proliferation and differentiation assessments into account. Over the years, it has been shown that the NPI is useful, also compared to other models [21, 22]. Despite the fact that the NPI was validated for FBC patients aged <70 [4], with a mean age of 54 [12], the NPI performed well in discriminating good/moderate and poor prognosis in this group of MBC patients, of which the patients were substantially older.

Adjuvant! Online was first reported in 2001 as a computer program calculating overall survival, as well as absolute treatment benefits from hormone therapy and chemotherapy for FBC patients based on the SEER data [7]. Adjuvant! Online has been validated for FBC in several European countries [8, 13, 23] and shown to perform rather well. Quintyne et al. correlated actual outcome to the NPI as well as Adjuvant! Online for a cohort in the Republic of Ireland and noticed underestimation for both prognostic tools. This was explained by ethnic differences between the SEER database (heterogeneous) and the Irish cohort (only Caucasians) and by other factors [22]. Prognostication by Adjuvant!Online for Asian breast cancer patients [24] as well as for women older than the age of 65 years and comorbidity filled out as “average for age” [25] shows overestimation. Unfortunately, calibration of Adjuvant! Online could not be done because Adjuvant! Online is validated for 10-year survival, and the mean follow-up in this study was 4.6 years.

Predict was developed in the United Kingdom and based on 5694 women diagnosed with breast cancer in East Anglia from 1999 to 2003 [9]. It was the first prognostication tool for early FBC patients including HER-2-status and “mode of detection”. The model is based on breast cancer-specific mortality and competing mortality modelled separately. Predict was validated for FBC patients in 2011 in the British Colombia Dataset and compared with Adjuvant! Online [10, 27]. Both provide accurate overall and BCSS estimates and prognosticate comparably for FBC patients [27]. Predict has also recently been validated for Asian FBC patients and showed reasonable discrimination (area under the ROC curve of 0,78 for 5-year and 0,73 for 10-year overall survival) [28]. Even so, in the present group of MBC patients, prognostication by Predict performs as well as Adjuvant! Online (Tables S2, S3). These models use additional features compared with MPI and NPI, like HER2-status, Ki67 and mode of detection, on the basis of which better prediction was expected. However, since the vast majority of MBC is HER2 negative, Ki67 low [29] and symptomatic (in absence of a screenings program for men), it is understandable that no differences were found between the models for the same 88 patients (Table S2). Calculations with the maximum amount of patients resulted in only slightly better C-indexes (Table S3). This is probably due to the relatively greater number of missing data per patient (Table S1) and predicted survival probabilities that could still be calculated by scoring unavailable features as “unknown”. Calibration of Predict (Fig. 5.) showed that the predicted overall survival rates were higher than the actual observed overall survival. Age (mean 66.4 years) and gender (life expectancy for women is higher than for men), as well as mainly “low risk” tumour characteristics [26] and the fair amount of unavailable data, could be an explanation for overestimation. The prognostic groups of MPI (good and poor) and NPI (good, moderate and poor) were defined and validated by the test itself. Adjuvant! Online and Predict provide continuous survival probabilities. These data were categorized into tertiles, which allows comparison of the results to the NPI, which is used more frequently and more recently than the MPI. The classifications differentiated well between “good” and “poor prognosis” as well as between “moderate” and “poor prognosis”, while differentiation between “good” and “moderate prognosis” was not as good. This is probably due to the small amount of patients. Although the numbers are small, the best comparison of the different prognostic tests is made by looking at C-indices calculated for the same 88 patients, due to the least missing data (Table S1). Because of the limited amount of patients and missing data, the confidence intervals of the C-indices are rather wide. Small differences in predictive value of the different predictive models are therefore unable to detect within this group of patients. Analyses were restricted to 5-year survival data because 10-year survival data might be strongly influenced by age-related (non breast cancer) causes of death. Disease-specific survival data would give insight into this, but unfortunately, were not available. Another disadvantage of this study was the absence of central review of pathology in 38 %, although the tumour features of the present group of MBC patients is representative as compared to literature [26]. In this study, the amount of Her2 positivity was only 2.3 % (1.1 % unknown) for the same 88 patients. Other studies also described low percentages of Her2 positivity [26, 29]. The 34 % of Her2 positivity described by Korde et al. [30] seems to be exceptionally high. Mean age was around 65, which is about 10 years older than FBC patients and also found by others [1, 31]. This older age might be the reason that only 18.1 % of the patients received adjuvant chemotherapy, while 36.7 % of the patients had one or more positive axillary lymph node(s).

Based on the wide time frame of our group of patients and the fact that the MPI and the NPI were derived many years ago, when treatments were considerably different and diagnostics not as sophisticated as today, one would expect difficulties in applying results obtained from these models in today’s care. However, all these models performed well in survival analysis, with comparable C-indexes and confidence intervals, indicating that there are no major differences in the performance of these models for MBC patients. Mook et al. reported C-indices of 0,71 for breast cancer-specific survival and 0,70 for overall survival in a cohort of 5380 women with primary breast cancer using Adjuvant! Online for prognostication. These C-indices are comparable to our group of men with breast cancer (Tables S2, S3).

In conclusion, the MPI, NPI, Adjuvant! and Predict prognostic models that were originally validated for FBC also perform quite well for MBC. Further improvements in MBC prediction may be expected from molecular studies [32, 33] and gene array.

## Electronic supplementary material

Below is the link to the electronic supplementary material.

Supplementary material 1 (TIFF 12745 kb)

Supplementary material 2 (TIFF 13156 kb)

Supplementary material 3 (TIFF 13177 kb)
