# Supplementary figures and images for: Prognostic models in male breast cancer
Source: Breast Cancer Res Treat. 2016 Sep 26;160(2):339–46. doi: 10.1007/s10549-016-3991-9 (PMC5065611; doi:10.1007/s10549-016-3991-9)

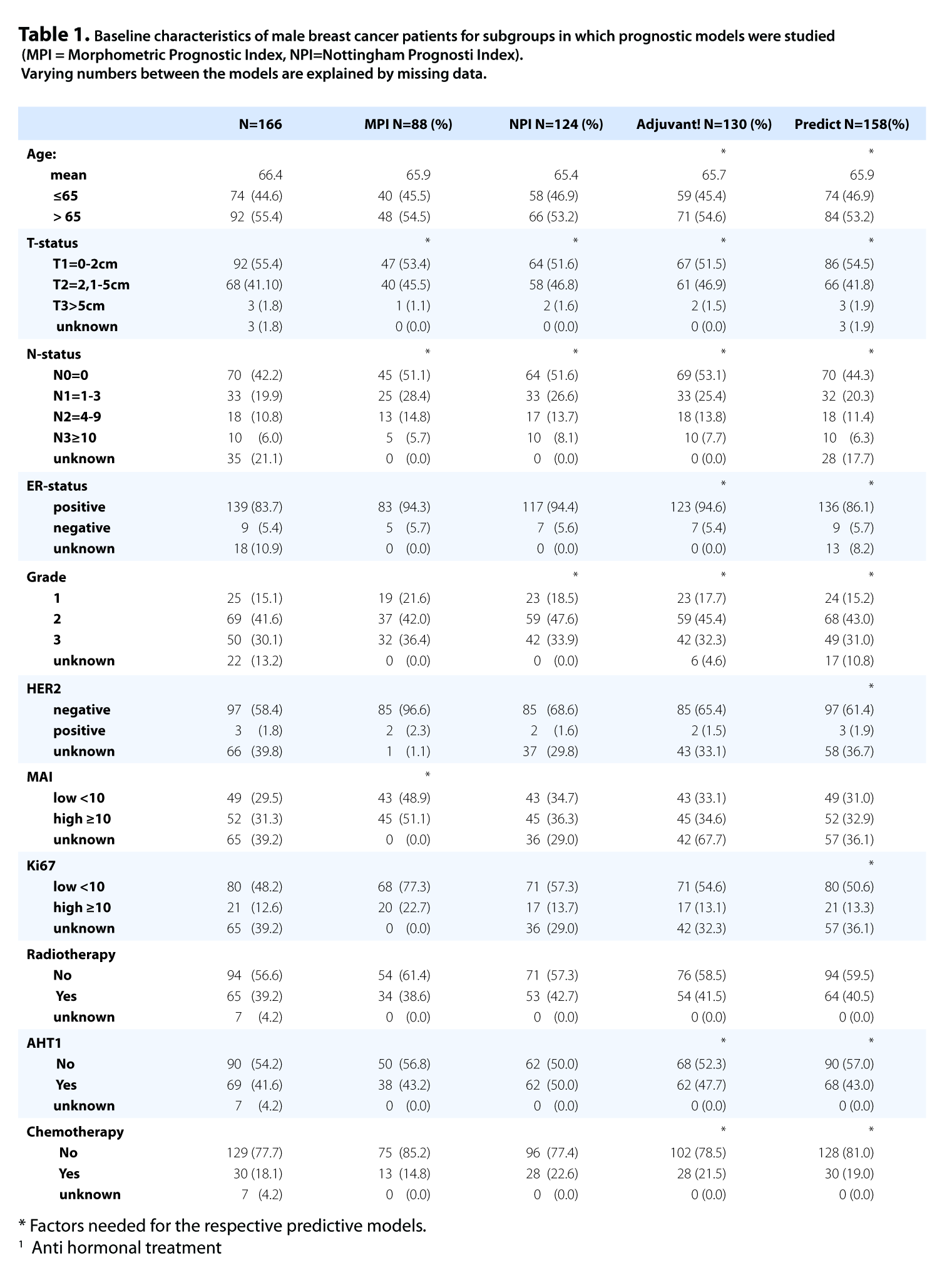

Supplement: Supplementary file 1 — Supplementary material 1 (TIFF 12745 kb) [file 10549_2016_3991_MOESM1_ESM.tif]

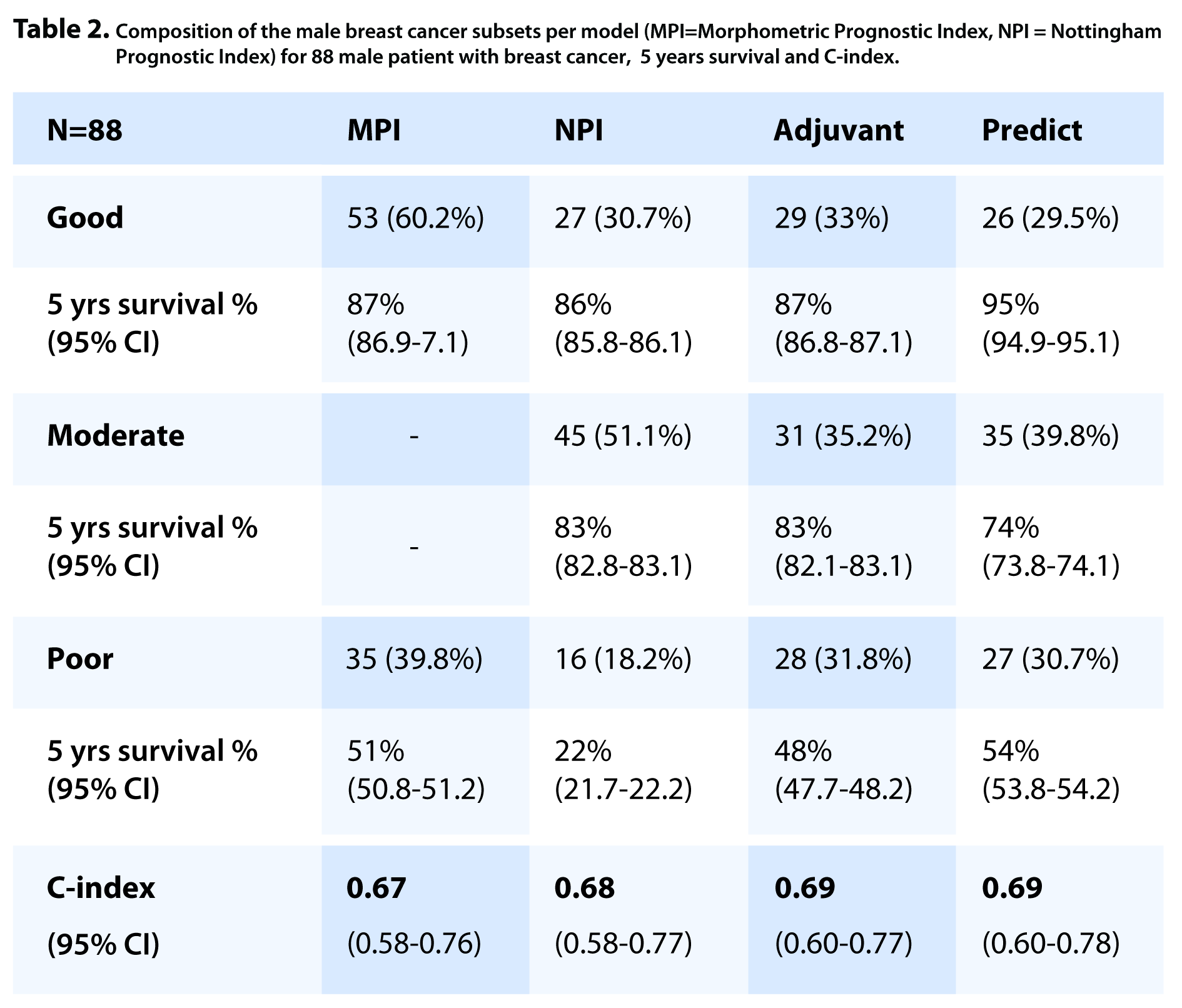

Supplement: Supplementary file 2 — Supplementary material 2 (TIFF 13156 kb) [file 10549_2016_3991_MOESM2_ESM.tif]

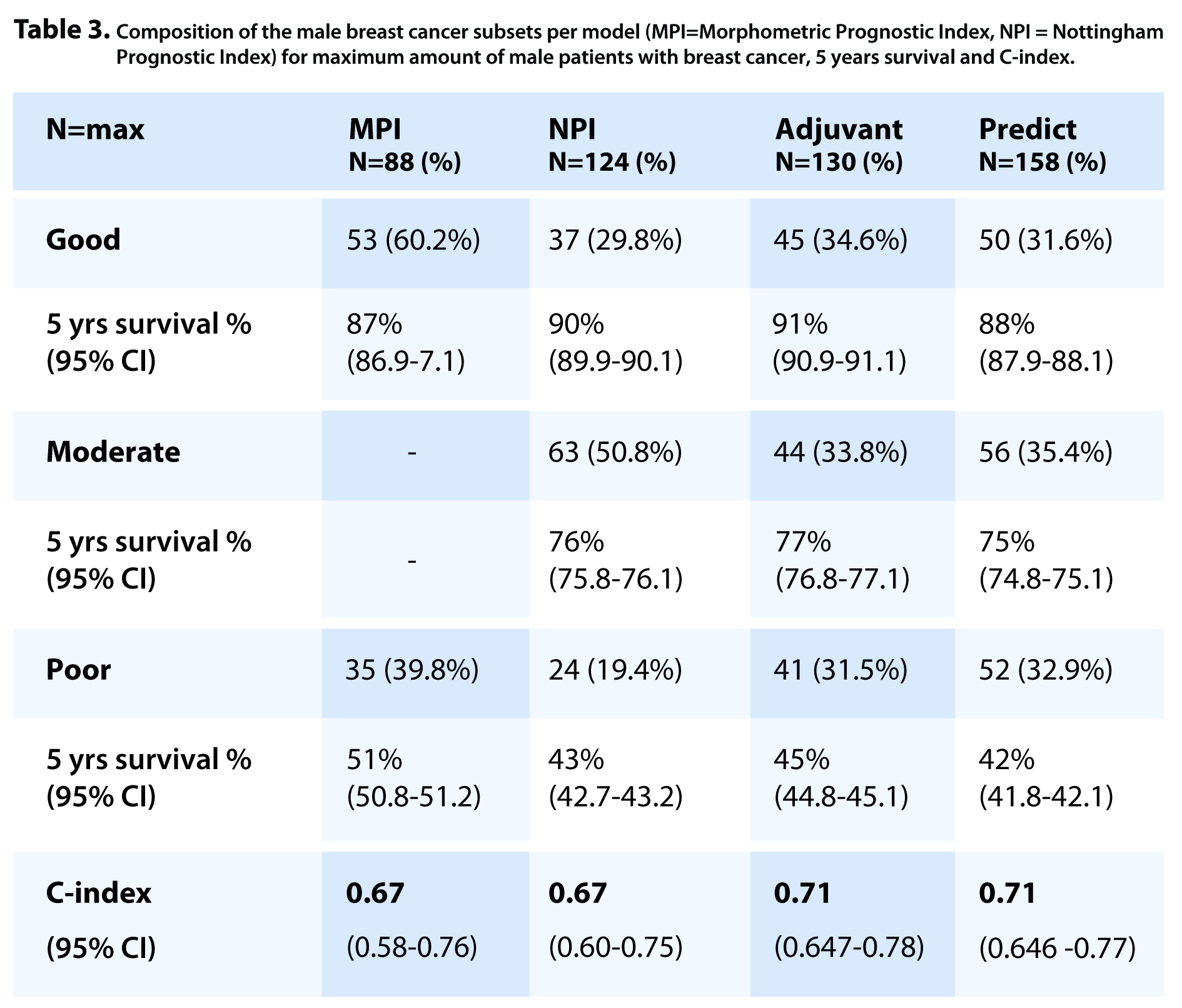

Supplement: Supplementary file 3 — Supplementary material 3 (TIFF 13177 kb) [file 10549_2016_3991_MOESM3_ESM.tif]
